# Supplementary material for: Causal Relationship between Aging and Anorexia Nervosa: A White-Matter-Microstructure-Mediated Mendelian Randomization Analysis
Source: Biomedicines. 2024 Aug 16;12(8):1874. doi: 10.3390/biomedicines12081874 (PMC11351342; doi:10.3390/biomedicines12081874)
Supplement: Supplementary file 1 [file biomedicines-12-01874-s001.zip › Supplementary Figure/Figure S4/Singe SNP plot of MR analysis for TL to ubm-b-2085.pdf]

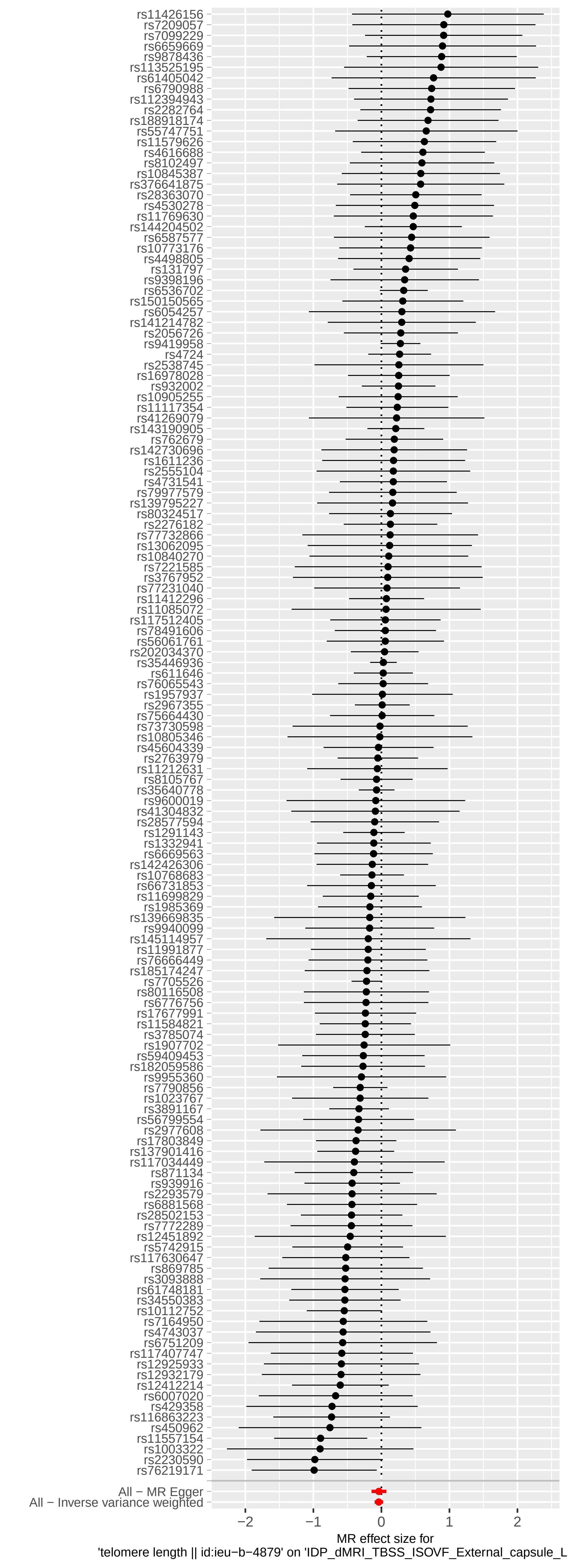
rs11426156
rs7209057
rs7099229
rs6659669
rs9878436
rs113525195
rs61405042
rs6790988
rs112394943
rs2282764
rs188918174
rs55747751
rs11579626
rs4616688
rs8102497
rs10845387
rs376641875
rs28363070
rs4530278
rs11769630
rs144204502
rs6587577
rs10773176
rs4498805
rs131797
rs9398196
rs6536702
rs150150565
rs6054257
rs141214782
rs2056726
rs9419958
rs4724
rs2538745
rs16978028
rs932002
rs10905255
rs11117354
rs41269079
rs143190905
rs762679
rs142730696
rs1611236
rs2555104
rs4731541
rs79977579
rs139795227
rs80324517
rs2276182
rs77732866
rs13062095
rs10840270
rs7221585
rs3767952
rs77231040
rs11412296
rs11085072
rs117512405
rs78491606
rs56061761
rs202034370
rs35446936
rs611646
rs76065543
rs1957937
rs2967355
rs75664430
rs73730598
rs10805346
rs45604339
rs2763979
rs11212631
rs8105767
rs35640778
rs9600019
rs41304832
rs28577594
rs1291143
rs1332941
rs6669563
rs142426306
rs10768683
rs66731853
rs11699829
rs1985369
rs139669835
rs9940099
rs145114957
rs11991877
rs76666449
rs185174247
rs7705526
rs80116508
rs6776756
rs17677991
rs11584821
rs3785074
rs1907702
rs59409453
rs182059586
rs9955360
rs7790856
rs1023767
rs3891167
rs56799554
rs2977608
rs17803849
rs137901416
rs117034449
rs871134
rs939916
rs2293579
rs6881568
rs28502153
rs7772289
rs12451892
rs5742915
rs117630647
rs869785
rs3093888
rs61748181
rs34550383
rs10112752
rs7164950
rs4743037
rs6751209
rs117407747
rs12925933
rs12932179
rs12412214
rs6007020
rs429358
rs116863223
rs450962
rs11557154
rs1003322
rs2230590
rs76219171
All − MR Egger
All − Inverse variance weighted
−2
−1
0
1
2
MR effect size for
'telomere length || id:ieu−b−4879' on 'IDP_dMRI_TBSS_ISOVF_External_capsule_L
